# Detection and Severity Scoring of Chronic Obstructive Pulmonary Disease Using Volumetric Analysis of Lung CT Images

**DOI:** 10.5812/iranjradiol.6759

**Published:** 2012-03-25

**Authors:** Mohammad Parsa Hosseini, Hamid Soltanian-Zadeh, Shahram Akhlaghpoor

**Affiliations:** 1 Department of Electrical and Computer Engineering, Science and Research Branch, Islamic Azad University, Tehran, Iran; 2 Image Analysis Laboratory, Department of Radiology, Henry Ford Health System, Detroit, MI, USA; 3 Control and Intelligent Processing Center of Excellence, School of Electrical and Computer Engineering, University of Tehran, Tehran, Iran; 4 Department of Radiology, Sina Hospital, Tehran University of Medical Sciences (TUMS), Tehran, Iran

**Keywords:** Pulmonary Disease, Chronic Obstructive, Diagnosis, Computer-Assisted, Tomography, X-Ray Computed, Lung

## Abstract

**Background:**

Chronic obstructive pulmonary disease (COPD) is a devastating disease.While there is no cure for COPD and the lung damage associated with this disease cannot be reversed, it is still very important to diagnose it as early as possible.

**Objectives:**

In this paper, we propose a novel method based on the measurement of air trapping in the lungs from CT images to detect COPD and to evaluate its severity.

**Patients and Methods:**

Twenty-five patients and twelve normal adults were included in this study. The proposed method found volumetric changes of the lungs from inspiration to expiration. To this end, trachea CT images at full inspiration and expiration were compared and changes in the areas and volumes of the lungs between inspiration and expiration were used to define quantitative measures (features). Using these features,the subjects were classified into two groups of normal and COPD patients using a Bayesian classifier. In addition, t-tests were applied to evaluate discrimination powers of the features for this classification.

**Results:**

For the cases studied, the proposed method estimated air trapping in the lungs from CT images without human intervention. Based on the results, a mathematical model was developed to relate variations of lung volumes to the severity of the disease.

**Conclusions:**

As a computer aided diagnosis (CAD) system, the proposed method may assist radiologists in the detection of COPD. It quantifies air trapping in the lungs and thus may assist them with the scoring of the disease by quantifying the severity of the disease.

## 1. Background

Chronic obstructive pulmonary disease (COPD) is characterized by airflow limitation that is not fully reversible. It is caused by a mixture of airway obstruction (bronchitis and bronchiolitis) and parenchymal destruction (emphysema), the relative contributions of which are variable [1]. This obstruction decreases the rate of airflow through the lungs when a person breathes out. In addition to bronchitis and emphysema, the result of irreversible airflow obstruction could have dominant features of asthma. An estimated 8-17% of American men and 10-19% of American women suffer from chronic airway obstruction [2]. In 1990, a study by the World Bank and the World Health Organization (WHO) ranked COPD 12th as a burden of disease. By 2020, it is estimated that COPD will be ranked 5th in the world and it is projected to be the third leading cause of death for both males and females in the US [3].

Pulmonary function tests are the primary diagnostic tools for COPD after the medical history and physical examination. The pulmonary function tests are difficult and time consuming and require physician involvement. Computed tomography has been the main imaging approach in lung diseases including COPD. With the older CT systems, only axial imaging was feasible,but high resolution 1 mm thick slices could be obtained at 1 cm increments to generate detailed images of the lung structure [4]. Evaluation of radiographic features may provide important diagnostic and prognostic information. In addition, changes in these features over time may be used to evaluate new treatment options and to monitor treatment responses [5][6]. However, assessment of COPD using CT has a number of potential limitations. For example, mild COPD may be missed on high resolution CT (HRCT). In addition, in patients with severe emphysema, the extent of destruction may be underestimated because localized areas of destruction may be invisible [7].

One of the major research subjects in medical imaging and diagnostic radiology is computer-aided diagnosis (CAD) systems. CAD systems can help to improve the diagnostic accuracy of radiologists, lighten the burden of increasing workload and assist them in the interpretation of medical images [8][9][10][11].

Success of the CAD systems depends on accurate segmentation of the lungs. In recent years, computer assisted segmentation of pulmonary CT images has been done through semi-automatic and automatic techniques. The number of segmentation algorithms found in the literature is very high [12]. The active contour models proposed by Kass, Xu and Prince [13][14] are extensively used in medical image processing applications and particularly to locate object boundaries. Several procedures have been proposed for improving this method through changes in snake forces and initialization [15][16][17][18].

## 2. Objectives

The aim of this study was to design a new CAD system to assess COPD in lung CT Images. The proposed CAD system is computationally efficient and relieves radiologists from the time-consuming task of COPD detection.

## 3. Patients and Methods

### 3.1. Patient Selection

**Figure 1:**
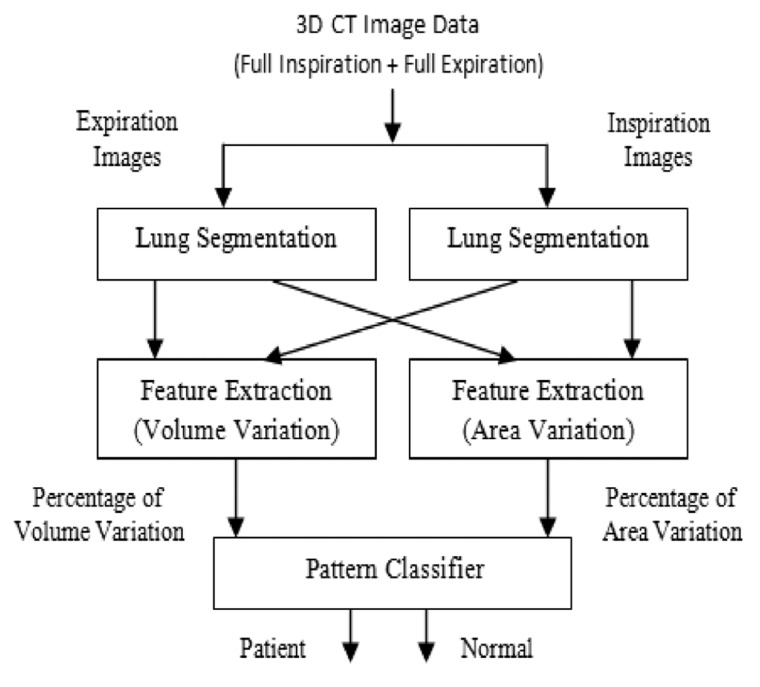
Diagram of the proposed method

Twenty-five patients (mean age, 59.7 years; range, 25-78 years) and twelve healthy subjects (mean age, 41.3 years; range, 22-53 years) were enrolled into the study. Totally, 56.7% of the subjects were male and the others were female. All of the subjects were imaged for their routine diagnosis process, so they were not exposed to additional radiation for this research. The normal group was selected from the patients who claimed they were contaminated with chemical agents during the Iran-Iraq war. These patients were introduced to the imaging clinic for further examinations. Comprehensive clinical examination, CT scan analysis, and respiratory tests revealed that those subjects had completely normal conditions, so they were selected as our normal population. As shown in Figure 1, our method consisted of three main steps: First, the extraction step to identify the lung regions. Second, the feature extraction step to find parenchyma variation in 2D and 3D, for finding and comparing elastic recoil. Finally, a pattern classifier for categorizing the subjects into normal subjects and patients was used.

### 3.2. CT Imaging

The thorax CT image sets were acquired at Noor Medical Imaging Center, Tehran, Iran using a SIEMENS HRCT scanner (Sensation 64). The voxel size was 1 × 1 × 3 mm. The CT data acquisition were, 120/0 kV, 254/0 mA and 1/0 mm. Scanning voltage was 120 kV and the current was 254 mA. In each case, CT of the thorax was performed from the lung apices through the level of the adrenal glands at full inspiration and was repeated at full expiration. The mean breath-hold was 7 seconds for one scan. All imaging was performed with a collimation of 16 × 1.25 mm, table feed of 30 mm/rotation and rotation time of 0.6 second/360° tube rotation with a standard reconstruction algorithm.

The scanner was subject to a weekly quality assessment with a phantom check including uniformity, linearity and noise. Air and water phantoms were used to calibrate the CT scanner.

### 3.3. Segmentation of Lungs

**Figure 2:**
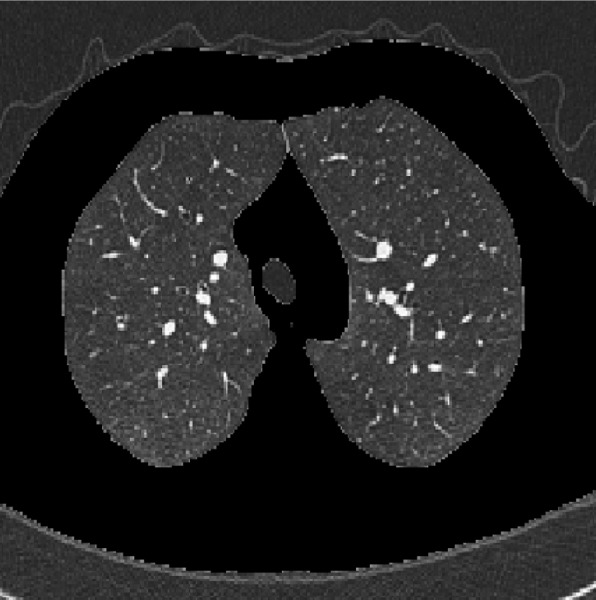
The result of segmentation on a cut at inspiration

To segment the lungs and extract appropriate ROI active contour methods were applied. In this case minimizing an energy function is used. The energy function consists of an external force calculated from the image data and an internal force defined from the curvature of the contour. Figure 2 shows the result of segmentation in one cut at inspiration.

### 3.4. Feature Extraction

COPD leads to a limitation of the flow of air to and from the lungs causing shortness of breath. Lung volumes and the amount of air-trapping, total lung capacity (TLC), residual volume (RV) and functional residual capacity (FRC) are related to the degree of hyperinflation of the lungs and are all characteristically increased in COPD [19], so the volumetric variations in 2D and 3D as area and volume variations are decreased in COPD. Increased residual volume signifies air trapping. This demonstrates an obstruction to exhalation.

### 3.5. Area Variation

**Figure 3:**
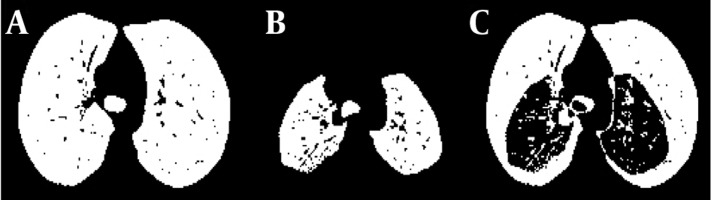
A, Lung area at inspiration in a healthy subject; B, Lung area at expiration; C, Difference between inspiration and expiration

**Figure 4:**
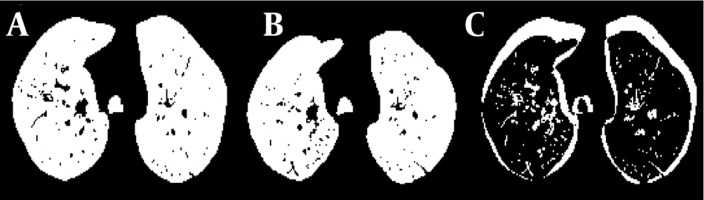
A, Lung area at inspiration in a patient; B, Lung area at expiration; C, Difference between inspiration and expiration

Here, changes in the areas of the lungs have been evaluated in two dimensions. To this end, only one cut of CT is analysed. For comparison of variation we needed to find corresponding images in each mode of respiration. First, we selected corresponding inspiration and expiration images based on anatomical features. Then we found the mask of lung in each state. To this end, we used the carina trachea cut in inspiration and expiration images. The carina is the apex of the bifurcation point of the trachea. On the other hand, carina trachea is the ridge separating the openings of the right and left main bronchi. It is usually located at the level of the 4th to 5th thoracic vertebrae [20]. For finding the area variation, we compared the numbers of each mask’s pixels. To remove the effects of age, height and sex, a normalized value can be used. For this approach the inspiration area was used as a reference parameter. The area variation data sets were divided by their inspiration areas in order to negate that variable’s effect on the data. This allows data on different scales to be compared, by bringing them to a common scale. Figure 3 shows this feature in a 22-year-old healthy woman and Figure 4 shows it in a 42-year-old female patient. The advantage of this approach was its fast calculation. Its disadvantages were assessing COPD only in one region of the lung and the need to find corresponding images at inspiration and expiration.

### 3.6. Volume Variation

**Figure 5:**
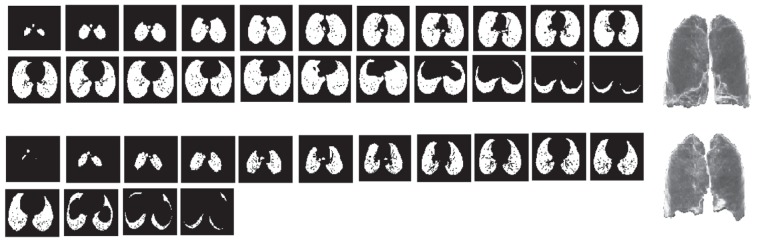
Different cuts through the lung at expiration and the same cuts at inspiration

Here, changes in the total lung volume in three dimensions were evaluated. The results are normalized to the lung volume at inspiration. Figure 5 shows the lung parenchyma at inspiration and the same cuts at expiration for a 22-year-old healthy female subject. To reduce computation time, one cut in every 17 cuts was used. Advantages of the method included accuracy and no need to find corresponding images at inspiration and expiration. Its disadvantage was its computational complexity.

### 3.7. Pattern Classifier

In practice, a radiologist may not be able to recognize small volumetric variations reliably from the CT images.Our purpose was to develop an automatic method to reduce labor-intensive procedures to detect air-trapping as a result of COPD or an indicator of the susceptible subject to get COPD. We used a pattern classifier to categorize our result into two classes of normal and patients subjects. Pattern classification is about assigning labels to objects. Objects are described by one or a set of measurements called features [21]. Our features were the area variation and volume variation. Accurate diagnosis depended on accurate classification of features to one of the normal or patient classes. The assumption of Gaussian distribution was used for the data set. Minimizing the probability of making an error was the goal. To this end, the Bayes rule to assign data sets to one of the two classes was used. We had two classes, ω_1_ and ω_2_ with normal distribution. The goal was to minimize the probability of making an error and there was no information regarding an object other than the class probability distribution.

Bayesian decision theory is a fundamental approach based on quantifying the tradeoffs between various classification decisions and the costs that accompany such decisions [22]. Our problem was a supervised classification. We had a set of data samples with associated labels, the class types. These were used as our samples in the Bayes classifier design. The Bayes rule for minimum error classification was: *l_r_(x)* = *p(x│ω_1_)* / *p(x│ω_2_) ⟩p(ω_2_)* / *p(ω_1_)⟹ x ∈ classω_1_*

Where *p* (x│ω_1_) was the class-conditional density function of class 1 and *p* (ω_1_) was the a priori probability. The function *l_r_(x)* set as the likelihood ratio.

## 4. Results

We now present the experimental results of applying the proposed methods on the clinical CT images. The pattern classifier found hard threshold for a decision that minimizes the Bayes classification risk. The Bayes decision threshold was 0.276 for the area variation and 0.310 for the volume variation.

**Figure 6:**
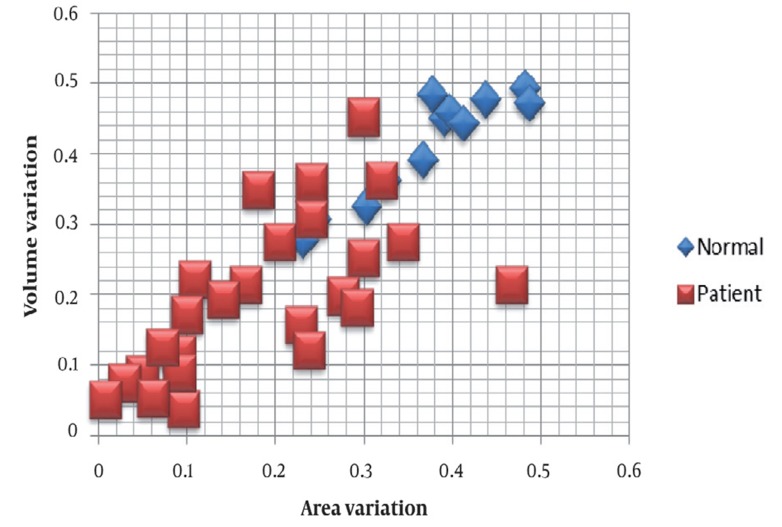
Scatter plot of the area and volume variations in the normal and patient classes

Scatter Plots were used to investigate possible relationships between the two features described above. To model the relationship between the area and volume variations, we used the least square method to draw the straight line of best fit. The best fitting for each class is shown in Figure 6. Equation {^Δ*V_N_* = 0.855Δ*S_N_* + 0.095^_(R_N_)^2^ = 0.859_ represents the best linear fits for the normal and equation {^ΔV_N_ = 0.626ΔS_N_ + 0.079^_(R_N_)^2^ = 0.424 _

fits for the normal and equation represents patient classes. Where ΔV_n_ is the volume variation,ΔS_n_ is the area variation, and (RN)2 indicates accuracy of regression.

### 4.1. Statistical Analysis

We used t-test to assess whether the means of the two groups of normal subjects and patients are statistically different. Table 1 shows mean and standard deviation (STDEV) of each class and Table 2 shows the result of t-test. The results of the Bayes classification are given in Tabulation as a misclassification matrix or cross tabulation between test and gold standard. The sensitivity, specificity, positive predictive value (PPV), negative predictive value (NPV) and the accuracy of our method were 84%, 83.3%, 91.3%, 71.4% and 83.8%, respectively.

**Table 1:** Mean and STDEV of the Features (Area and Volume Variation)

**Group**	**Sample Size**	**Feature 1**		**Feature 2**	
		**Mean **	**STDEV a**	**Mean**	**STDEV**
Normal	12	0.3711	0.0825	0.4121	0.0762
Patient	25	0.1749	0.2341	0.1996	0.1981

^a^ Abbreviation: STDEV, standard deviation

**Table 2:** The t-Test Results

**Feature**	***t*-Test Value**	**Degrees of Freedom**	***P *value**	**Different Between Means**
Area variation	5.0648	19.8192	3.041e-005	yes
Volume variation	5.1078	18.3338	3.480e-005	yes

**Tabulation:** Cross Tabulation Between Test and Gold Standard

	Confusion Matrix	True Class	
		Positive (Patient)	Negative (Normal)
Test outcome (by Bayesian Method)	Positive	21	2
	Negative	4	10

## 5. Discussion

Without using image analysis results, radiologists are limited in their sensitivity, specificity, and diagnostic accuracy. Due to many parenchymal structures, it is sometimes extremely difficult to decide whether or not a HRCT is abnormal. A CAD approach to the diagnosis of lung diseases can be helpful. In addition, a computerized method is needed to monitor the success or failure of treatment. In this paper, we presented a new CAD system for identification of air trapping in the lungs in COPD. This method finds the lung parenchyma at inspiration and expiration in the corresponding CT images and their difference is calculated. For removing the effect of age, weight and height, the differences are normalized. This method can be used in 2D to evaluate area variation and in 3D to evaluate volume variation. The 3D method estimates the lung variation more accurately and does not need identification of the corresponding images. The proposed method can be used for scoring the severity of air-trapping in the lung for the diagnosis and treatment evaluation as well as screening individuals at the risk of COPD.

Our study was different from other studies in the materials and methods. Hosseini et al. proposed a new scheme for evaluation of air-trapping based on CT images [23]. The proposed method finds volumetric variations of the lungs from inspiration to expiration states. Hosseini et al. suggested a novel method for identification of COPD using CT images in separated lungs [24]. The changes in three important cuts that show air trapping more than other cuts are evaluated in this method. In another study, some abnormalities of the shape of lung fields in chest radiographs are used as indicators of emphysema [25].

Our study had limitations. Intervention is sometimes necessary to ensure a full inspiration. Another limitation of this technique was related to the effects of age on the lung elasticity. To overcome this limitation, at least partially, normalized patterns were used. To this end, extracted features of CT images were normalized to the inspiration area as a reference.

The experimental results showed that the system could extract surface and volume variations satisfactorily and the analyzed results outputted from the system were useful for quantitative diagnosis of COPD with a classifier.

In conclusion, CAD has become a part of clinical work in the detection of some diseases such as breast cancer by use of mammograms, but it is still in the infancy of its applications in lung diseases such as asthma and bronchitis.While there is no cure for COPD and the lung damage that results in this disease cannot be reversed, it is very important to diagnose it as early as possible. In this paper,we proposed a new CAD system which may be helpful to radiologists for a more accurate and early diagnosis.
